# Metabolic Risk Factors at the Time of Cancer Diagnosis in Primary Care: A Cross-Sectional Study

**DOI:** 10.7759/cureus.87086

**Published:** 2025-07-01

**Authors:** Katarzyna Brukało, Magda Szostak, Jerzy Slowik

**Affiliations:** 1 Department of Health Policy, School of Public Health, Medical University of Silesia, Bytom, POL; 2 Department of Health Promotion and Community Nursing, Faculty of Health Sciences, Medical University of Silesia, Katowice, POL; 3 Department of Family Medicine, "Familia" Group Practice of Family Physicians, Siemianowice Śląskie, POL

**Keywords:** cancer prevention, early detection, metabolic risk factors, primary care, public health

## Abstract

Background

Cancer is a growing public health burden and a leading cause of death and disability globally. An estimated 30-50% of cancer deaths are preventable through the modification of behavioral and metabolic risk factors. Primary care offers an essential platform for population-level cancer prevention and early detection, especially by leveraging existing screening and chronic disease monitoring infrastructure. This study, conducted in a single primary care clinic in Poland, evaluates metabolic markers routinely collected in this setting to assess their potential role in identifying cancer risk.

Methods

We conducted a cross-sectional study analyzing 347 adult patients diagnosed with cancer (ICD-10 codes: C00-C14, C15-C26, C30-C39, C43-C44, C50, C51-C58, C60-C63, C64-C68, C69-C72, C73-C75, C76-C80, C81-C96, D00-D48) between 2017 and 2022 in a single primary care clinic in Poland. Fasting glucose, total cholesterol, triglycerides, and body mass index (BMI) were selected because they are routinely measured in primary care settings and frequently associated with metabolic risk and cancer development. These parameters were analyzed in relation to cancer type using descriptive statistics, chi-square tests, and Kruskal-Wallis non-parametric analyses.

Results

Elevated fasting glucose (≥100 mg/dL) was significantly associated with cancer type (p = 0.0238). This abnormality was observed in 83.6% (N = 290) of all patients. Although BMI, cholesterol, and triglyceride levels were not significantly associated with cancer type, metabolic abnormalities were highly prevalent in the entire sample. A total of 89.1% (N = 309) of patients were overweight or obese, and nearly one-third (N = 110) presented with all four metabolic abnormalities concurrently. Only 2.3% (N = 8) of the patients had none of the measured metabolic risk factors. The vast majority, 97.7% (N = 339), had at least one metabolic abnormality. Given the high prevalence of metabolic disturbances, the possibility of selection bias should be considered, as individuals visiting primary care may be more likely to have pre-existing chronic conditions or risk factors under surveillance.

Conclusions

Routine, low-cost metabolic tests performed in primary care may offer useful insights into cancer risk patterns and could inform opportunistic risk stratification efforts. However, given the cross-sectional design of this study, the findings do not support causal inference. Integrating routinely collected metabolic data into public health frameworks may enhance early detection strategies and support timely diagnostic referrals. These results highlight the potential for scalable, population-based interventions within primary care to contribute to reducing the cancer burden, morbidity, and disability-adjusted life years.

## Introduction

Cancer is a major public health challenge worldwide, consistently ranked as the second leading cause of death. In 2022, approximately 9.7 million cancer-related deaths occurred globally - nearly one in six deaths [1]. The burden of cancer continues to grow not only in terms of mortality but also in terms of years of healthy life lost. According to the World Health Organization, in 2021, cancer accounted for 8.8% of global disability-adjusted life years - a measure that combines years of life lost due to premature death and years lived with disability [2]. Importantly, an estimated 30-50% of cancer deaths are preventable through the reduction of modifiable risk factors and implementation of early detection strategies [2]. Given the strong links between metabolic disturbances and cancer, we hypothesized that selected metabolic markers - body mass index (BMI), fasting glucose, total cholesterol, and triglycerides - would vary significantly across different cancer types at the time of diagnosis, reflecting their potential utility in opportunistic risk stratification in primary care.

Primary health care (PHC) offers a unique opportunity for early intervention in cancer prevention and detection. As the first point of contact for most individuals, primary care settings are positioned to carry out comprehensive, continuous, and person-centered services that integrate clinical, behavioral, and social dimensions of health [3]. This includes monitoring risk factors and lifestyle behaviors that may contribute to cancer development. The recent World Health Organization guidance emphasizes the importance of integrating noncommunicable disease (NCD) prevention and cancer screening within PHC to improve early detection, reduce health inequities, and strengthen population-level interventions.

A range of metabolic and lifestyle-related factors, particularly overweight, obesity, impaired glucose metabolism, and lipid abnormalities, have been well-documented as contributors to cancer risk [4-6]. These factors are closely tied to NCDs such as type 2 diabetes and cardiovascular disease, but their oncological implications have gained increasing attention. For instance, obesity and hyperglycemia are associated with elevated levels of insulin and insulin-like growth factors, which can promote tumor initiation and progression by stimulating cellular proliferation and inhibiting apoptosis [6]. Chronic low-grade inflammation, commonly present in obesity, may also create a pro-tumorigenic microenvironment. Similarly, dyslipidemia, although less consistently associated with cancer risk, may contribute to oxidative stress and lipid peroxidation, both of which can induce DNA damage and promote carcinogenesis. Together, these metabolic disturbances may influence cancer development through hormonal, inflammatory, and immunological pathways [7].

Crucially, these markers - BMI, fasting glucose, total cholesterol, and triglycerides - are already monitored in many primary care settings, particularly in the context of chronic disease management. Their availability and low cost make them attractive for potential use in opportunistic cancer risk stratification. However, it is important to acknowledge that cancer is a multifactorial disease, and these metabolic indicators represent only a subset of relevant risk factors. Genetic predisposition, environmental exposures, infections, and other determinants also play critical roles in cancer development and should be considered when interpreting the potential of these markers [3].

The aim of this study was to evaluate the relationship between these basic metabolic parameters and the type of cancer diagnosed in patients attending a single primary care clinic. By analyzing a population of 347 patients with newly diagnosed malignancies, we sought to determine whether these routinely available indicators could be associated with specific tumor types and potentially serve as tools to support earlier identification of oncological risk in a public health context.

## Materials and methods

This cross-sectional observational study was conducted in a single primary care clinic in Poland and included adult patients (aged ≥18 years) who received a new diagnosis of cancer between 2017 and 2022. While this setting allowed for detailed examination of routinely collected metabolic data at the point of diagnosis, the findings may have limited generalizability beyond this clinic and region. Differences in population characteristics, health system structures, and cancer diagnostic pathways across regions and countries should be considered when interpreting the results. The study aimed to assess whether basic metabolic parameters - BMI, fasting glucose, total cholesterol, and triglycerides - differed significantly across cancer types at the time of diagnosis. The analysis was based on a retrospective review of anonymized electronic medical records. In accordance with national regulations, ethical approval was not required.

Eligible patients were those with a confirmed first-time diagnosis of malignant or in situ neoplasms, as coded by ICD-10, with available fasting blood test results (glucose, total cholesterol, triglycerides) and anthropometric data (height and weight) for BMI calculation. Patients with missing laboratory or anthropometric data were excluded.

Extracted variables included fasting glucose (mg/dL), total cholesterol (mg/dL), triglycerides (mg/dL), and BMI (kg/m²). BMI was calculated as weight divided by height squared. Patients were further categorized by age (18-49, 50-59, 60-69, 70-79, 80-89, and ≥90 years), sex (male or female), and cancer type. Cancer types were grouped into diagnostic categories based on ICD-10 codes (e.g., digestive organs, breast, male genital organs, female genital organs, urinary tract, hematopoietic/lymphoid tissues, respiratory system). The specific ICD-10 codes and their corresponding cancer categories are presented in Table 1.

**Table 1:** ICD-10 codes used for grouping cancer types into diagnostic categories

Cancer type	ICD-10
Benign neoplasm or of unspecified/unknown nature	D00–D48
Malignant neoplasm of male genital organs	C60–C63
Malignant neoplasm of digestive organs	C15–C26
Malignant neoplasm of indefinite, secondary, and unspecified location	C76–C80
Malignant neoplasm of the eye, brain, and other parts of the central nervous system	C69–C72
Malignant neoplasm of the breast	C50
Malignant neoplasm of the skin (including melanoma)	C43–C44
Malignant neoplasm of the thyroid and other endocrine glands	C73–C75
Malignant neoplasm of the lymphoid, hematopoietic, and related tissue	C81–C96
Malignant neoplasm of the urinary tract	C64–C68
Malignant neoplasm of the respiratory and intrathoracic organs	C30–C39
Malignant neoplasm of the lip, mouth, and throat	C00–C14
Malignant neoplasm of female genital organs	C51–C58

Each metabolic parameter was classified as “normal” or “abnormal” based on widely accepted clinical reference values derived from international guidelines. BMI was considered normal if between 18.5 and 24.9 kg/m²; values ≥25 kg/m² (overweight or obese) or <18.5 kg/m² (underweight) were classified as abnormal, in accordance with the World Health Organization classification [8]. Fasting glucose was classified as normal if <100 mg/dL and abnormal if ≥100 mg/dL, following the American Diabetes Association criteria for impaired fasting glucose [9]. Total cholesterol was considered normal if ≤190 mg/dL, consistent with recommendations of the European Society of Cardiology (ECS) for cardiovascular prevention [10]. Triglycerides were classified as normal if <150 mg/dL and abnormal if ≥150 mg/dL, based on cut-offs used in ESC/European Atherosclerosis Society dyslipidemia guidelines [10].

Descriptive statistics were calculated for all variables. Means, medians, standard deviations (SD), and interquartile ranges (IQRs) were used for continuous data, while categorical variables were summarized as frequencies (N) and percentages (%). No formal sensitivity analyses were conducted; however, subgroup distributions were reviewed to ensure consistency of results across major cancer categories. Between-group comparisons for continuous metabolic parameters across cancer types were conducted using the Kruskal-Wallis test due to non-normal data distribution. Where relevant, pairwise comparisons were performed using the Mann-Whitney U test. Associations between categorical variables (e.g., number of abnormal risk factors by cancer group) were tested using the chi-square test (χ²). A p-value of <0.05 was considered statistically significant. No adjustments for multiple comparisons were applied, as the analyses were exploratory and hypothesis-generating. All analyses were performed using SPSS Version 27 (IBM Corp., Armonk, NY).

## Results

The study cohort included 347 patients diagnosed with cancer between 2017 and 2022 in a primary care setting. Among these patients, 161 (46.4%) were women and 186 (53.6%) were men. The most common age groups were 60-69 years (28.8%, N = 100) and 70-79 years (28.0%, N = 97), jointly comprising more than half of the sample. A detailed breakdown of age and sex distribution is presented in Table 2.

**Table 2:** Age and sex distribution of the study population (N = 347)

Age group (years)	Female (N)	Male (N)	Total (N)	% of total
18–49	17	10	27	7.8%
50–59	26	13	39	11.2%
60–69	44	56	100	28.8%
70–79	35	62	97	28.0%
80–89	34	36	70	20.2%
90+	5	9	14	4.0%
Total	161	186	347	100%

The most frequently observed cancer types were male genital organ cancers (22.5%, N = 78; ICD-10: C60-C63), digestive organ cancers (13.5%, N = 47; C15-C26), breast cancer (11.2%, N = 39; C50), and urinary tract malignancies (12.1%, N = 42; C64-C68). Other cancer types included tumors of the female genital organs (5.5%, N = 19; C51-C58), lymphoid and hematopoietic tissues (6.3%, N = 22; C81-C96), and cancers of the lip, mouth, or throat (1.4%, N = 5; C00-C14). It should be noted that, as this study was conducted within a single primary care clinic, the cancer types observed may reflect the specific referral and diagnostic patterns of that setting. This may introduce a degree of selection bias, as patients seen in primary care may differ from the general population in terms of cancer presentation, comorbidities, or healthcare-seeking behavior.

A detailed summary of descriptive statistics for BMI, fasting glucose, total cholesterol, and triglycerides by cancer type is presented in Table 3.

**Table 3:** Descriptive statistics of metabolic parameters by cancer type (N = 347) Data are presented as mean ± SD or median [IQR/min–max] for each parameter, grouped by diagnostic category

Cancer type	ICD-10 codes	N	BMI, mean ± SD	Glucose, median [IQR]	Cholesterol, mean ± SD	Triglycerides, median [min–max]
Benign neoplasm or of unspecified/unknown nature	D00–D48	49	30.43 ± 4.84	107.1 [95.0–118.0]	199.6 ± 42.2	141.0 [49.0–346.0]
Malignant neoplasm of male genital organs	C60–C63	78	29.62 ± 2.60	108.5 [100.0–115.2]	194.0 ± 24.0	145.0 [67.0–335.0]
Malignant neoplasm of digestive organs	C15–C26	47	29.32 ± 4.27	107.7 [94.3–116.6]	191.9 ± 34.6	129.0 [54.0–463.0]
Malignant neoplasm of indefinite, secondary, and unspecified location	C76–C80	27	29.59 ± 4.71	104.8 [94.3–112.7]	204.3 ± 44.5	148.0 [54.0–301.0]
Malignant neoplasm of the eye, brain, and other parts of the central nervous system	C69–C72	3	29.65 ± 4.57	101.5 [96.0–104.0]	192.0 ± 32.8	155.0 [101.0–161.0]
Malignant neoplasm of the breast	C50	39	29.96 ± 6.95	104.4 [94.9–119.2]	207.3 ± 54.5	126.0 [54.0–287.0]
Malignant neoplasm of the skin (including melanoma)	C43–C44	6	28.18 ± 4.88	100.0 [91.0–120.0]	199.2 ± 55.4	122.5 [91.0–196.0]
Malignant neoplasm of the thyroid and other endocrine glands	C73–C75	5	29.61 ± 2.56	102.0 [96.0–109.0]	197.8 ± 29.1	132.0 [79.0–178.0]
Malignant neoplasm of the lymphoid, hematopoietic, and related tissue	C81–C96	22	30.01 ± 2.74	106.0 [103.3–109.5]	196.5 ± 45.8	137.5 [72.0–228.0]
Malignant neoplasm of the urinary tract	C64–C68	42	29.58 ± 3.10	107.9 [98.3–126.3]	193.8 ± 36.8	165.0 [36.0–222.0]
Malignant neoplasm of respiratory and intrathoracic organs	C30–C39	5	28.72 ± 2.85	102.0 [99.0–107.0]	208.0 ± 43.1	149.0 [99.0–192.0]
Malignant neoplasm of the lip, mouth, and throat	C00–C14	5	32.72 ± 5.95	108.6 [103.8–114.5]	188.6 ± 20.8	136.0 [94.0–164.0]
Malignant neoplasm of female genital organs	C51–C58	19	29.43 ± 4.47	110.3 [104.8–130.0]	225.2 ± 55.1	158.0 [17.2–353.0]

With regard to nutritional status, only 33 (9.5%) patients had a normal BMI (18.5-24.9 kg/m²), while the vast majority, 309 (89.1%) patients, were either overweight or obese. The highest mean BMI was observed in patients with cancers of the lip, mouth, and throat (32.7 kg/m²), followed by hematopoietic malignancies (30.0 kg/m²) and breast cancer (30.0 kg/m²). However, the Kruskal-Wallis test did not identify a statistically significant difference in BMI across cancer types (p = 0.0986).

Abnormal fasting glucose levels (≥100 mg/dL) were recorded in 290 (83.6%) patients. The highest mean fasting glucose was found in patients with cancers of the female genital organs (110.3 mg/dL) and urinary tract (107.9 mg/dL), while patients with breast cancer exhibited the lowest average values (104.4 mg/dL). A statistically significant association was observed between fasting glucose level and cancer type (p = 0.0238), suggesting a potential link between glycemic dysregulation and certain malignancies. While effect sizes such as odds ratios were not applicable due to the use of continuous variables and the non-parametric analytic approach, this result warrants further investigation using multivariable or longitudinal models.

Total cholesterol exceeded 190 mg/dL in 200 (57.6%) patients, with the highest mean values among patients with female genital cancers (225.2 mg/dL) and breast cancer (207.3 mg/dL). However, cholesterol levels were not significantly associated with cancer type (p = 0.5261).

Triglyceride levels were elevated (≥150 mg/dL) in 154 (44.4%) patients. The highest mean triglyceride values were found in patients with urinary tract (165.0 mg/dL) and female genital cancers (158.0 mg/dL), while the lowest values were observed in breast cancer cases (126.0 mg/dL). Similar to cholesterol, no statistically significant differences were found across cancer types for triglycerides (p = 0.7950).

When analyzing the cumulative burden of metabolic abnormalities, only eight (2.3%) patients presented with none of the four analyzed risk factors, namely abnormal BMI, fasting glucose, total cholesterol, and triglycerides. A total of 39 (11.2%) patients exhibited one abnormal parameter, while 75 (21.6%) had two, and 115 (33.1%) had three. Notably, 110 (31.7%) patients had all four metabolic abnormalities concurrently. In total, 339 (97.7%) out of 347 patients had at least one metabolic risk factor at the time of cancer diagnosis.

Metabolic syndrome (MetS) is a clinical construct describing the co-occurrence of multiple metabolic abnormalities, such as abdominal obesity, hyperglycemia, dyslipidemia, and hypertension, that collectively increase the risk of cardiovascular disease and some cancers. In this study, although there were minor differences in the number of concurrent metabolic risk factors among the different cancer groups, these differences were not statistically significant (p = 0.3967). Clustering of three or more metabolic abnormalities was common across both sexes, observed in 122 (65.8%) of 185 men and 102 (63.4%) of 161 women. When stratified by age, the proportion of patients with ≥3 metabolic risk factors increased from 16 (59.3%) of 27 in the age group of 18-49 years to 58 (59.8%) of 97 among those aged 70-79 years. Although a general trend toward higher clustering with increasing age was observed, no statistically significant differences were found across sex or age groups. A summary of the distribution of patients by number of concurrent metabolic abnormalities is presented in Table 4.

**Table 4:** Distribution of concurrent metabolic risk factors among cancer patients (N = 347)

Number of risk factors	No. of patients	Percentage (%)
0	8	2.3
1	39	11.2
2	75	21.6
3	115	33.1
4	110	31.7

Most patients had two or three abnormal results. Although the average number of risk factors per patient varied slightly among cancer types, no statistically significant differences were found (p = 0.3967).

Tables 5-8 present a comparative analysis of metabolic parameters across cancer types. For each cancer group, median values and IQRs are provided for BMI, fasting glucose, total cholesterol, and triglycerides. Statistical comparisons using the Kruskal-Wallis test showed that only glucose levels varied significantly across cancer types (p = 0.0238). No statistically significant differences were found for BMI (p = 0.0986), total cholesterol (p = 0.5261), or triglycerides (p = 0.7950). These results suggest that, despite the high prevalence of metabolic abnormalities at diagnosis, their distribution was largely consistent across the spectrum of malignancies analyzed.

**Table 5:** Comparison of BMI (kg/m²) across cancer groups p = 0.0986 (p-value calculated using the Kruskal–Wallis test)

Cancer group	ICD-10 codes	Median	IQR
Benign, in situ and neoplasms of uncertain or unknown behavior	D00–D48	28.40	3.52
Malignant neoplasms of male genital organs	C60–C63	30.05	1.85
Malignant neoplasms of digestive organs	C15–C26	28.30	4.75
Malignant neoplasms of ill-defined, secondary, and unspecified sites	C76–C80	28.60	3.93
Malignant neoplasms of the eye, brain, and other parts of the central nervous system	C69–C72	29.45	2.6
Malignant neoplasm of the breast	C50	29.40	6.2
Malignant neoplasms of the skin (including melanoma)	C43–C44	26.50	5.4
Malignant neoplasms of the thyroid and other endocrine glands	C73–C75	30.40	9.25
Malignant neoplasms of the lymphoid, hematopoietic, and related tissue	C81–C96	30.45	3.18
Malignant neoplasms of the urinary tract	C64–C68	29.45	3.03
Malignant neoplasms of the respiratory system and intrathoracic organs	C30–C39	28.60	5.05
Malignant neoplasms of the lip, oral cavity, and pharynx	C00–C14	29.60	4.0
Malignant neoplasms of female genital organs	C51–C58	30.10	4.70

**Table 6:** Comparison of fasting glucose (mg/dL) across cancer groups p = 0.0238 (p-value calculated using the Kruskal–Wallis test)

Cancer group	ICD-10 codes	Median	IQR
Benign, in situ and neoplasms of uncertain or unknown behavior	D00–D48	105.21	6.37
Malignant neoplasms of male genital organs	C60–C63	108.52	6.52
Malignant neoplasms of digestive organs	C15–C26	107.65	21.12
Malignant neoplasms of ill-defined, secondary, and unspecified sites	C76–C80	105.89	6.98
Malignant neoplasms of the eye, brain, and other parts of the central nervous system	C69–C72	106.87	5.35
Malignant neoplasm of the breast	C50	104.41	11.87
Malignant neoplasms of the skin (including melanoma)	C43–C44	108.97	2.17
Malignant neoplasms of the thyroid and other endocrine glands	C73–C75	106.40	14.38
Malignant neoplasms of the lymphoid, hematopoietic, and related tissue	C81–C96	106.03	3.61
Malignant neoplasms of the urinary tract	C64–C68	107.84	13.87
Malignant neoplasms of the respiratory system and intrathoracic organs	C30–C39	103.39	9.65
Malignant neoplasms of the lip, oral cavity, and pharynx	C00–C14	108.62	5.80
Malignant neoplasms of female genital organs	C51–C58	110.31	22.93

**Table 7:** Comparison of total cholesterol (mg/dL) across cancer groups p = 0.5261 (p-value calculated using the Kruskal–Wallis test)

Cancer group	ICD-10 codes	Median	IQR
Benign, in situ and neoplasms of uncertain or unknown behavior	D00–D48	183.50	45.25
Malignant neoplasms of male genital organs	C60–C63	196.00	19.75
Malignant neoplasms of digestive organs	C15–C26	198.00	55.06
Malignant neoplasms of ill-defined, secondary, and unspecified sites	C76–C80	230.55	68.25
Malignant neoplasms of the eye, brain, and other parts of the central nervous system	C69–C72	183.50	27.0
Malignant neoplasm of the breast	C50	203.00	84.05
Malignant neoplasms of the skin (including melanoma)	C43–C44	205.00	23.6
Malignant neoplasms of the thyroid and other endocrine glands	C73–C75	216.00	54.25
Malignant neoplasms of the lymphoid, hematopoietic, and related tissue	C81–C96	200.00	44.0
Malignant neoplasms of the urinary tract	C64–C68	198.00	23.75
Malignant neoplasms of the respiratory system and intrathoracic organs	C30–C39	193.50	36.75
Malignant neoplasms of the lip, oral cavity, and pharynx	C00–C14	187.00	11.0
Malignant neoplasms of female genital organs	C51–C58	229.00	83.9

**Table 8:** Comparison of triglycerides (mg/dL) across cancer groups p = 0.7950 (p-value calculated using the Kruskal–Wallis test)

Cancer group	ICD-10 codes	Median	IQR
Benign, in situ and neoplasms of uncertain or unknown behavior	D00–D48	144.00	85.0
Malignant neoplasms of male genital organs	C60–C63	145.00	32.0
Malignant neoplasms of digestive organs	C15–C26	129.00	80.0
Malignant neoplasms of ill-defined, secondary, and unspecified sites	C76–C80	158.50	71.25
Malignant neoplasms of the eye, brain, and other parts of the central nervous system	C69–C72	145.00	41.0
Malignant neoplasm of the breast	C50	126.00	76.0
Malignant neoplasms of the skin (including melanoma)	C43–C44	111.00	93.5
Malignant neoplasms of the thyroid and other endocrine glands	C73–C75	131.00	66.5
Malignant neoplasms of the lymphoid, hematopoietic, and related tissue	C81–C96	137.50	44.25
Malignant neoplasms of the urinary tract	C64–C68	165.00	50.75
Malignant neoplasms of the respiratory system and intrathoracic organs	C30–C39	133.00	62.0
Malignant neoplasms of the lip, oral cavity, and pharynx	C00–C14	136.00	45.0
Malignant neoplasms of female genital organs	C51–C58	158.00	87.0

## Discussion

The findings of our study indicate that a considerable proportion of patients are diagnosed with cancer while presenting with metabolic abnormalities such as elevated BMI, hyperglycemia, or lipid disorders, which are typically associated with MetS. This supports existing evidence linking MetS to increased cancer risk, particularly for gastrointestinal, gynecological, and genitourinary malignancies [11-13]. However, our results should be interpreted with caution given the inherent limitations of the study design. The cross-sectional and single-center nature of the analysis restricts causal inference and generalizability. Additionally, unmeasured confounding factors such as smoking, alcohol use, genetic predisposition, and physical activity may have influenced the observed associations.

Multiple systematic reviews and meta-analyses have confirmed that MetS increases the risk of developing several types of cancer, including colorectal [12], endometrial [13], breast [14], hepatocellular [15], and pancreatic cancers [16]. Other studies have demonstrated similar associations for renal [17], bladder [18], and ovarian cancers [19]. This clustering of metabolic risk factors may have a synergistic carcinogenic effect mediated by hyperinsulinemia, chronic inflammation, oxidative stress, and altered sex hormone levels [20].

The link between MetS and cancer has substantial public health implications, particularly given the rising global prevalence of obesity, type 2 diabetes, and associated metabolic dysfunctions. In recent decades, the prevalence of MetS has increased in parallel with lifestyle changes and population aging. Global data indicate that cancer deaths attributable to metabolic risk factors (e.g., high BMI, hyperglycemia) have more than doubled from 1990 to 2019, reaching an estimated 865,000 deaths annually - a 167% increase over three decades [21]. However, the high prevalence of metabolic abnormalities observed in our study sample may also reflect alternative explanations, such as referral or selection bias, regional variations in population health, or increased detection due to evolving diagnostic practices in primary care. These factors should be considered when interpreting the findings, particularly given the single-center design and limited geographic scope.

These findings underscore the need to integrate metabolic disease prevention with cancer control strategies. Weight loss and glycemic control, through lifestyle modification, pharmacotherapy (e.g., GLP-1 receptor agonists), or bariatric surgery, have been shown to reduce the risk of obesity-related and diabetes-associated cancers [22-24]. For instance, a sustained ≥10% weight loss has been linked to a significant reduction in cancer incidence among individuals with obesity and prediabetes or diabetes [22]. As such, interventions targeting metabolic health could provide dual benefits for cardiovascular and oncological outcomes. Nevertheless, prospective studies are needed to validate these findings, establish temporal relationships, and determine whether metabolic markers can serve as reliable predictors of cancer risk in diverse populations.

Given the limited resources in healthcare systems, combining metabolic disease management with cancer prevention may be an efficient approach. Recognition of MetS as a cancer risk factor expands the scope of chronic disease prevention and highlights the need for interdisciplinary collaboration among endocrinologists, oncologists, public health professionals, and primary care providers.

Primary care settings offer a unique opportunity for both the detection of MetS and the early identification of cancer risk. Our study, which was conducted in a primary care context, reveals that many patients had unaddressed metabolic disorders at the time of cancer diagnosis, representing potentially missed opportunities for prevention or earlier detection. These findings suggest a need to consider the integration of routine metabolic screening into standard primary care workflows as part of opportunistic cancer prevention efforts. Given that tests such as BMI, fasting glucose, and lipid profiles are already widely used and relatively low-cost, especially compared to imaging or tumor markers, such an approach may be feasible and cost-effective even in resource-constrained settings. Incorporating these data into cancer risk assessment tools could support more timely referrals and targeted interventions, particularly for populations at elevated baseline risk.

Since primary care professionals routinely monitor BMI, glucose levels, and lipid profiles, they are well positioned to identify patients at elevated oncologic risk. Patients with MetS should receive tailored counseling on their cancer risk and be encouraged to adopt healthier lifestyles. Moreover, they should undergo all recommended age-appropriate screenings and, in some cases, may benefit from enhanced surveillance strategies. For example, individuals with MetS or type 2 diabetes are at an elevated risk of colorectal cancer, prompting some experts to recommend earlier or more frequent colonoscopy in this high-risk group [12].

Furthermore, metabolic abnormalities may serve as early indicators of occult malignancy. A prime example is new-onset type 2 diabetes in older adults, which has been identified as an early signal of pancreatic cancer. Studies show that 30-50% of pancreatic cancer patients develop new-onset diabetes within three years prior to diagnosis [25]. New-onset diabetes in this age group increases pancreatic cancer risk two- to threefold [25]. Consequently, there are calls to introduce targeted cancer screening, such as pancreatic imaging, for patients with unexplained, abrupt glycemic deterioration.

The emerging concept of a "cardio-renal-metabolic-cancer" (CRMC) continuum supports a more integrated clinical framework, encouraging metabolic risk assessment during cancer work-up and vice versa. In practice, this means patients with new-onset diabetes or MetS in primary care should also undergo careful cancer risk assessment and timely screening for malignancies such as breast, prostate, and colorectal cancer. Recent integrative reviews highlight the bidirectional interactions between cardiovascular, renal, metabolic, and oncological health. They describe mechanisms, such as chronic inflammation, insulin resistance, oxidative stress, and treatment-related toxicities, in cancer patients that underpin this multisystem syndrome and its clinical relevance [26].

This study has several limitations. First, it was based on data from a single primary care clinic, which may limit the generalizability of the findings to other populations or healthcare settings. Future research should include multi-center or population-based studies with larger, more diverse cohorts to improve external validity and explore regional or systemic differences in cancer-metabolic risk clustering. Second, the retrospective design relies on the accuracy and completeness of electronic medical records, which may introduce information bias. Third, due to the cross-sectional nature of the study, causality between metabolic abnormalities and cancer development cannot be established. Finally, the lack of information on cancer staging, treatment history, and long-term outcomes prevents deeper insights into prognostic implications. Despite these limitations, the study provides a valuable snapshot of the coexistence of metabolic disorders and cancer at the point of diagnosis in primary care and underscores the need for further prospective, multicenter research.

## Conclusions

This study highlights a high prevalence of metabolic risk factors among patients newly diagnosed with cancer in primary care, particularly overweight/obesity and elevated fasting glucose. The statistically significant association between glucose levels and cancer type suggests that glycemic disturbances may be an important marker for oncologic risk stratification.

Given the routine availability of metabolic data in primary care, these findings support the integration of basic metabolic profiling into early detection strategies. Identifying patients with multiple concurrent abnormalities may facilitate timely referrals and preventive interventions, contributing to reduced cancer burden and improved public health outcomes. However, these findings should be viewed as preliminary. Further prospective and interventional research is needed to validate the utility, safety, and cost-effectiveness of implementing metabolic risk-based cancer screening before its adoption into routine clinical practice.
